# Wischnewsky spots in starved dogs and cats and their relevance in veterinary forensic pathology

**DOI:** 10.1177/03009858261445993

**Published:** 2026-05-12

**Authors:** Francesca Maccagnan, Ana S. Gomes, Hannah McLaughlin, Alyson Blanchard, Sean Taylor, Lorenzo Ressel, Emanuele Ricci

**Affiliations:** 1 Istituto Zooprofilattico Sperimentale del Lazio e della Toscana “M. Aleandri,” Grosseto, Italy; 2 University of Trás-os-Montes and Alto Douro (UTAD), Vila Real, Portugal; 3 University of Liverpool, Neston, UK; 4 University of Salford, UK

**Keywords:** cats, cachexia, dogs, starvation, stomach, Wischnewsky spots

## Abstract

In 1895, the German pathologist Dr S.M. Wischnewsky described black, plaque-like discolorations of the gastric mucosa in humans whose cause of death was associated with hypothermia. The pathogenesis of these gastric lesions, known as Wischnewsky spots, is thought to be associated with blood redistribution mechanisms as the body attempts to preserve core body temperature in hypothermic conditions. However, the complete pathogenesis is not fully understood. The relationship between Wischnewsky spots and hypothermia in nonhuman species has also been documented, but again, such lesions in veterinary pathology have an unclear pathogenesis. This study investigated the presence of Wischnewsky spots in several veterinary forensic cases involving 89 emaciated dogs and 46 emaciated cats submitted to the Department of Veterinary Pathology, University of Liverpool, from 2016 to 2023. The gastric lesions were categorized, and collected data were used to compare the characteristics of those lesions found within animals deemed to have died from starvation compared with those having died via cachexic mechanisms. This study aimed to further understand the pathogenesis and diagnostic significance of Wischnewsky spots in veterinary forensic cases. The results indicated that Wischnewsky spots have a significant association with cats and dogs deemed to have died by starvation compared with cases of cachexia. Although comprehensive pathogenesis remains unclear, veterinary forensic cases with Wischnewsky spots are likely to be linked to the combination of stress and hypothermia experienced by cats and dogs that die because of starvation.

Among the numerous eponyms in medical literature, the term Wischnewsky spot (WS) is well established in pathology. It refers to plaque-like, black discolorations of the gastric mucosa, strongly associated with hypothermia.^8,18,23,27,30^ The eponym traces its origin to the German pathologist S.M. Wischnewsky,^29^ who first described these lesions in 1895 following autopsies on individuals who had succumbed to cold exposure.

In forensic practice, identifying WSs is a critical marker for hypothermia, particularly so when environmental conditions or contextual evidence suggest cold exposure.^7^ Their diagnostic utility is enhanced when combined with other indicators, such as body posture, clothing, and ambient temperature.^23^ Grossly, WSs appear as multiple small, round, dark brown to black petechiae, up to a few millimeters in diameter, flat, superficial, gastric mucosal lesions. The histopathological features of the WSs are the presence of small submucosal hemorrhages, vascular congestion, and fading of epithelial outlines, without apparent thrombosis and frank ulceration.^27^ Most importantly, no signs of active inflammation are discerned at the interface with normal gastric mucosa.^7,18,26^ The forensic significance of WSs lies in their association with hypothermia.^26,30^ These lesions likely result from ischemic changes in the gastric mucosa, precipitated by hypothermia-induced redistribution of blood flow. As body temperature declines, peripheral and splanchnic circulation is reduced to preserve core temperature, leading to mucosal ischemia, hemorrhage, and necrosis, manifesting as the characteristic black lesions observed during autopsy.^21^

Recent investigations have expanded the relevance of WSs beyond human forensic pathology to veterinary cases. These lesions have been identified in domestic animals,^24^ including dogs,^25^ rabbits,^20^ Japanese quails,^12^ and a brown howler monkey,^1^ suggesting a similar pathophysiological response to severe cold exposure. For instance, the presence of these spots in canine gastric mucosa underscores their potential value as markers for hypothermia-related deaths in veterinary forensic cases. Although less extensively documented in animals, these findings highlight the utility of WSs in diagnosing fatal hypothermia across species.

As an alternative or complementary pathogenetic mechanism for the formation WSs in humans, the occurrence of acute hemorrhages within the lamina propria of the gastric mucosa leads to the lysis of the extravasated erythrocytes with percolation of hemoglobin that, once in contact with the acid glandular secretion, will turn black (hematinization). In addition, the physiological response to cold stress in hypothermia is increasing the secretion of gastrin from endocrine G cells, pepsin from chromaffin cells, and histamine from enterochromaffin cells,^21^ facilitating the formation of WSs by boosting acid secretion and small mucosal hemorrhages.^30^

Animal studies have revealed intriguing results. For example, experiments in rats exposed to cold demonstrated that mitigating stress significantly reduced the size and number of gastric lesions, possibly by decreasing gastric acid secretion. This suggests that stress may act as an effect modifier, with hypothermia-induced lesions being exacerbated under conditions of heightened stress.^5^

Although these lesions are frequently seen in deaths caused by environmental hypothermia, they can also be seen in cases where hypothermia is not implicated. For example, WSs are also detected in diabetic ketoacidosis, to the point of speculating a common shared pathogenetic pathway, such as the damage of engorged mucosal capillaries leading to superficial hemorrhages and mucosal devitalization.^6^

This present study explores the presence of WSs in a cohort of dogs and cats that died from starvation, comparing their occurrence with cases of animals experiencing similar levels of emaciation due to chronic diseases leading to cachexia. By elucidating the comparative prevalence of these lesions, we aim to further contextualize the diagnostic value of WSs in both human and veterinary forensic investigations.

## Materials and Methods

### Study Population

The archive of cases submitted for postmortem examination at the Department of Veterinary Pathology at the University of Liverpool from 2016 to 2023 was searched for dogs and cats presenting with a body score inferior to 3 out of 9 (World Small Animal Veterinary Association body score)^13^ and compatible with poor body conditions and emaciation. Criteria for inclusion comprised: a complete postmortem examination with both detailed gross (all organs except the spinal cord) and histological examinations (2 paired appendicular muscles with related nerves and 19 organs or their segments) and a set of photos documenting the condition of the gastric mucosa following removal of the gastric content and gentle rinsing.

For each of the selected animals, the final postmortem report was scrutinized to detect the presence of concurrent diseases and to assign the emaciated animal to either starvation^10^ (no concurrent disease) or cachexia (concurrent disease) groups.

For each animal, the presence of edible and nonedible ingested material, the presence and number of WSs, the preferential location of WSs across the mucosa (pylorus, body, or fundus), and the presence of black discoloration of the gastric and intestinal contents were recorded. In addition, when available, the body retrieval location (indoor/outdoor) was recorded for each animal.

To obtain a semiquantitative scoring for the number of WSs in each animal, stomach samples were divided into 4 groups: 0, no WSs; 1, up to 5 discrete spots; 2, up to 10 discrete spots; and 3, gastric mucosa with disseminated pin-point spots or a large single discrete spot (>2 cm in diameter).

### Statistical Analysis

Statistical analyses were performed using IBM SPSS Statistics (Version 29) and R 4.4.0 (R Core Team, 2024). A Chi-square test of independence and corresponding effect size (Cramér’s V) were used to study associations between WSs and (1) condition (starvation/cachexia), (2) presence of foreign gastric material, and (3) where the animal was found (indoor/outdoor). Because the minimum expected count did not meet the threshold for a Chi-square test and because the marginal distributions of the contingency table were not fixed, Barnard’s exact test^3^ using the Barnard’s unconditional test package^15^ was chosen to examine associations between WSs and (1) black discoloration of intestinal content and (2) black gastric fluid.

## Results

One hundred and fifty-three dogs and 94 cats were retrieved following the initial database search. However, 64 dogs and 48 cats were excluded from the study because high-quality, close-up gross images of the rinsed gastric mucosa were unavailable, leaving a final total of 89 dogs and 46 cats for analysis. Applying the inclusion criteria (as stated previously) according to the species and condition resulted in 40 starvation cases and 49 cachectic cases in dogs, and 19 starvation cases and 27 cachectic cases in cats (Table 1).

**Table 1.** Summary of frequencies and percentages of pathological findings in starved and cachectic dogs and cats.

	Dogs		Cats	
	Starved	Cachectic	Starved	Cachectic
Total number	40	49	19	27
Average age (years ± SD)	5 (±4)	10 (±5)	2 (±3)	7 (±6)
Outdoor (%)	11 (30)	0	0	0
Foreign gastric material (%)	19 (47)	7 (14)	8 (42)	1 (4)
Edible gastric material (%)	18 (45)	32 (65)	7 (37)	8 (30)
Wischnewsky spots (%)	27 (67)	1 (2)^a^	8 (42)	1 (4)^b^
Prevalent distribution of WS (%)	Pyloric antrum (56)	Body (100)	Body (75)	Pyloric antrum (100)
Black gastric fluid (%)	23 (58)	0	2 (11)	0
Black discoloration of intestinal content (%)	24 (60)	1 (2)	6 (32)	2 (7)

Abbreviation: SD, standard deviation; WS, Wischnewsky spots.

a Multisystemic T-cell lymphoma with hypercalcemia of malignancy.

b Systemic toxoplasmosis.

**Figure 1.**
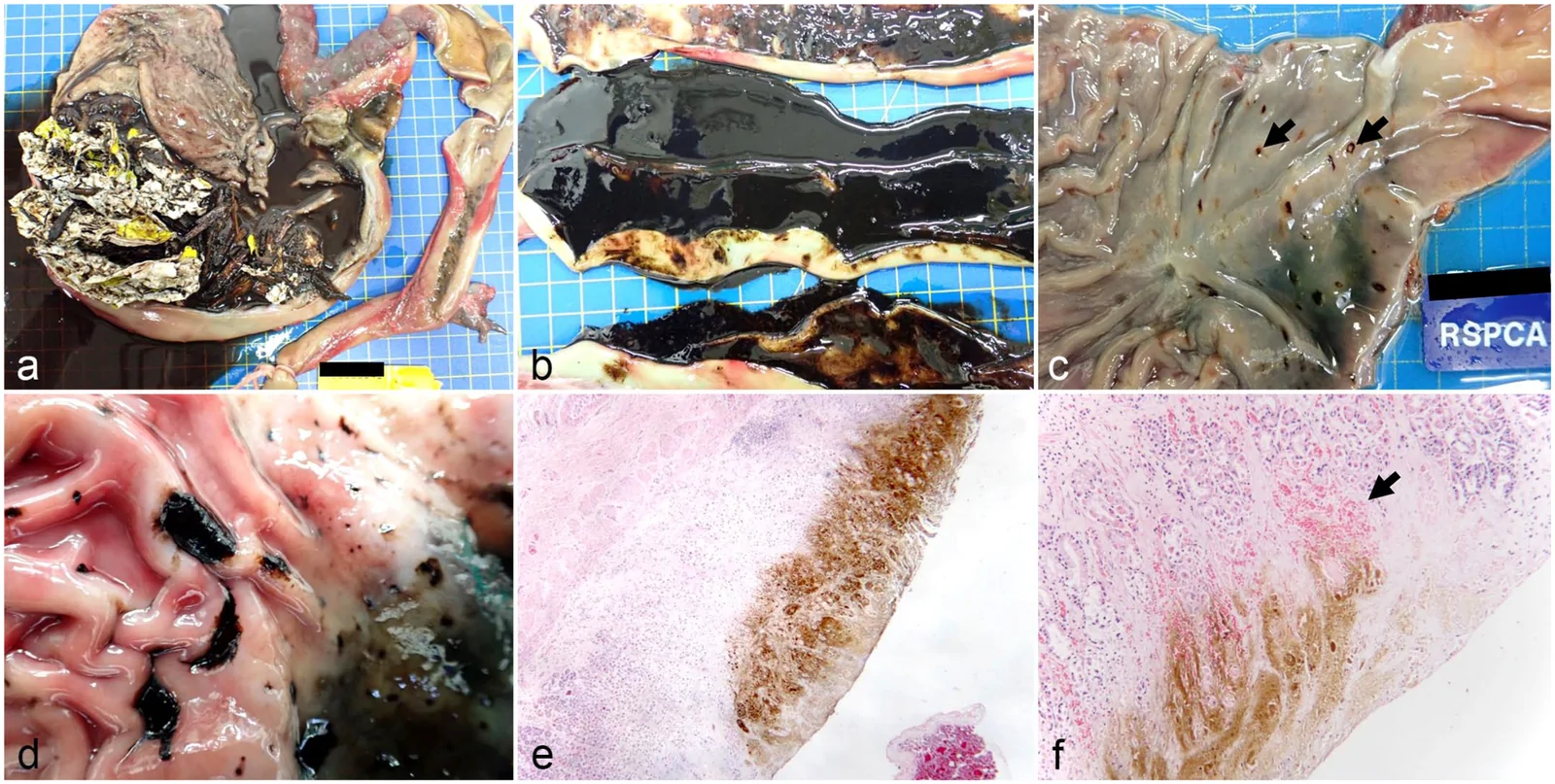
Gross and histological appearance of Wischnewsky spots (WSs) in starved dogs and associated findings. (a) Stomach and stomach contents. Crushed and folded aluminum, hard plastic, wood, and grass are surrounded by black fluid. (b) Jejunum upon incision containing black fluid. (c) Pyloric region of the gastric mucosa with myriads of flat black WSs (score 3, arrows). (d) Cluster of large, black, well-demarcated WSs of the gastric folds. (e) Stomach. Microscopic appearance of a single, discrete WS. Note the focal and superficial nature of the spot, which appears golden brown in hematoxylin and eosin-stained sections. Hematoxylin and eosin (HE). (f) Higher magnification of the WS in (e) showing a clear transition between extravasated erythrocytes (arrow) and more superficial, granular extracellular material (acid hematin). HE.

In 19 out of 40 starved dogs (47.5%) and 8 out of 19 starved cats (42%), nonedible foreign gastric material, such as tinfoil, fabric, and hard plastic, were noted (Fig. 1a). Regardless of the contemporaneous presence of ingested edible material and associated with the detection of WS, the lumina of the stomach and intestine contained abundant black fluid interpreted as a consequence of oxidized hemoglobin (Fig. 1a, b).

In our cases, WSs appeared as a single or small cluster of flat, plaque-like, round, oval, and rarely linear foci of dark brown to black discoloration of the mucosa, only infrequently appearing slightly concave (erosion). WSs were most prevalently noted over the pyloric region in dogs (56%) and the gastric body (75%) in cats and were often noted over the apex of gastric folds (Fig. 1c, d).

Histologically, WS were visible as discoid demarcated foci of granular golden discoloration, obscuring the silhouette of the gastric mucosa, which otherwise appeared devitalized, with only incidental observation of concurrent hemorrhage, as only rarely intact erythrocytes were discerned within or around a WS. Mucosal devitalization appeared either as full-thickness or superficial (luminal half of the mucosa) but never corresponded to an area of frank mucosal ulceration (Fig. 1e, f).

In both dogs and cats, the age of animals in the starvation group (dogs, average 5 ± 4 years; cats, average 2 ± 3 years) was lower than that of animals in the cachexia group (dogs, average 10 ± 5 years; cats, average 7 ± 6 years). Whereas both starved and cachectic cats were retrieved from sheltered, closed, vacated premises, 11 out of 40 starved dogs (30%) were found outside, often in garden sheds or courtyards.

WS were noted in 27 out of 40 starved dogs (67%) and in 8 out of 19 starved cats (42%), while only one animal presented WSs in the cachectic group, for both dogs (1 out of 49, 2%) and cats (1 out of 27, 4%). There was a significant, robust, association between WS and cases of starvation in dogs, χ^2^(1, N = 89) = 43.76, *p* < .001, *ϕc* = .70. Similarly, a moderate association between WS and cases of starvation was also observed in cats, χ^2^(1, N = 46) = 10.45, *p* < .001, *ϕc* = .48. When the number of WSs was assessed semiquantitatively (Table 2), both dogs and cats showed a prevalence of 1 to 5 small spots (score 1) over the gastric mucosa.

**Table 2.** Wischnewsky spots (WSs) scoring in starved and cachectic dogs and cats.

	Dogs		Cats	
	Starved (%)	Cachectic (%)	Starved (%)	Cachectic (%)
Score 0	13 (32)	48 (98)	11 (58)	26 (96)
Score 1	15 (38)	—	5 (26)	—
Score 2	6 (15)	1 (2)	2 (11)	1 (4)
Score 3	6 (15)	—	1 (5)	—

Ingested foreign gastric material was noted in 19 out of 40 starved dogs (47%) and 8 out of 19 starved cats (42%). The latter was also observed in 7 of 49 (14%) cachectic dogs and 1 of 27 (4%) cachectic cats. A significant association was also found between WS and the presence of foreign gastric material in dogs, χ^2^(1, N = 89) = 7.86, *p* = .005, *ϕc* = .30, but not in cats, χ^2^(1, N = 46) = 0.46, *p* = .50. Furthermore, a significant and moderate association was observed between the location of the retrieval of the carcase and WS, χ^2^(1, N = 87) = 12.58, *p* < .001, *ϕc* = .42, such that WS were more often present in starved dogs kept outside (2 cases were omitted from the analysis because their location was unknown). A Barnard’s exact test revealed a significant association between black gastric fluid and black discoloration of the intestinal contents in starvation cases, but not in cachexia cases, in dogs only (*T* = −8.19, *p* < .001). The association between WS and black discoloration of intestinal content was significant in both dogs (*T* = −9.00, *p* < .001, 2-tailed) and cats (*T* = −4.34, *p* < .001, 2-tailed), as was the association between WS and black gastric fluid content in dogs (*T* = −9.05, *p* < .001, 2-tailed) and cats (*T* = −2.93, *p* = .038, 2-tailed). Furthermore, there was no evidence of a sex predisposition for either condition or species, χ^2^(1, N = 122) = 0.60, *p* = 0.44.

## Discussion

WS are crucial in forensic pathology for indicating hypothermia as a cause of death. This study reported the first documentation of WSs and black discoloration of the semifluid gastrointestinal content in dogs and cats that died of starvation, comparing their prevalence with the stomach mucosa of dogs and cats that died following the terminal progression of natural long-term diseases capable of determining a comparable loss of body condition (cachexia group). These spots, observed over the gastric mucosa of the examined dogs and cats, ranged in color from brown to black and varied in size. The WSs in our study population presented identical macro and microscopic morphological features as those described in confirmed cases of hypothermia in both humans^2,7,27^ and animal cases.^1,12,24,25^

In a natural context, during the seasonal drop in food availability, a subset of animal species respond by adjusting their metabolism, leading to a state of hibernation, a physiological adaptation intended to reduce overall metabolism.^1^ The marked drop in body temperature is one of the key features of the above strategy, which evolved to reduce nutrient consumption during periods of minimal or zero nutrient availability.^4^ Even in experimental conditions, a drug-induced decrease in body temperature did not affect the rate of starvation-related death, likely indicating that the term hibernation better describes a more complex, yet harmonized, metabolic downregulation to reduce nutrient consumption in animals evolved to overcome starvation, safely. Thus, hypothermia is often the simple consequence of a marked reduction in thermogenic capacity in starved animals incapable of hibernation rather than a safeguarding strategy against excessive energy consumption.^22^

Animals undergoing starvation are known to develop hypoglycemia, anticipating a decrease in both fat and muscle mass, which then leads to a state of hypothermia.^9,19^ Interestingly, experimental studies in mice indicated that muscle mass loss via autophagy and a drop in body temperature occurred more severely in males than in females.^14^ On the contrary, ketone production was heightened by fasting in female mice, likely indicating a selective metabolic switch in estrogen-primed individuals, preferring fat mobilization (including from brown fat) over muscle gluconeogenesis for energy production, which also prevented a more rapid drop in body temperature than males. In our case series, we did not observe sex prevalence in either condition or species. This may be because our cohort of animals consisted of those that reached a fatal and terminal stage of starvation, following a long period of complete caloric restriction, whose duration remains unknown.

Among the few reports of WS in animal postmortem examinations,^1,24,25^ similarly to humans, the presence of WS has been documented and associated with the likelihood of death by hypothermia due to adverse environmental circumstances.

In our case series, the high prevalence of WS in dogs and cats was primarily associated with fatal starvation. Animals were at their terminal stages of muscle and adipose tissue mass depletion without alternative concurrent pathological conditions that could explain such a drastic metabolic shift toward global catabolism, other than the absence of available/administered food. In agreement with a previous study and as an indirect sign of preserved appetite,^10^ our population of starved dogs and cats frequently presented nonedible foreign material instead of nutritious ingesta within the otherwise empty stomach (allotriophagia). Even in the absence of ingested foreign material and in strong association with the presence of WS over the gastric mucosa, the content was otherwise represented by dark brown to often black fluid. Similarly, the scant intestinal content also displayed a characteristic black discoloration, which, under light microscopy, appeared composed of granular, golden material (hematinized hemoglobin), consistent with the gross interpretation of oxidized hemoglobin and sharing identical morphological and tinctorial affinities with the granular, golden material defining the WS of the gastric mucosa. Furthermore, histology of intestinal sections (duodenum, jejunum, ileum, and colon) never revealed hemoglobin or blood leakage from the mucosa. Thus, the WSs were deemed the source of luminal hemoglobin in the distal gastrointestinal tract.

In an experimental model in which rats were exposed to cold temperatures, younger animals showed a lower prevalence of WSs.^16,21^ Thus, age may have played a role in the lower incidence of WSs in our cohort of starved cats (average 2 years) compared with starved dogs (average 5 years). Furthermore, all starved cats were found deceased in confined spaces, rooms and premises without access to the outside, thus limiting their ability to escape and hunt. Conversely, approximately one-third of starved dogs were discovered outside, in small courtyards or enclosed gardens. Even if a statistical association was observed in our study between starved dogs retrieved outdoors and the presence of WSs, all starved cats and some starved dogs with WS were retrieved deceased indoors. The above observation challenges the exclusive role of external environmental factors in the causation of hypothermia with WSs.

Our findings indicate that starvation-induced death is frequently associated with WS formation on the gastric mucosa of dogs (67%) and cats (42%), preferentially at the level of the pyloric antrum (dogs) and gastric body (cats). While the formation of the WS is not interpreted as an effect of mechanical abrasion of the mucosa by ingested foreign material, the exact pathogenesis of WS formation in starvation remains elusive. Due to the retrospective nature of this study, direct measurement of the body or environmental temperatures was not possible to better quantify the drop in core body temperature. Still, we hypothesize that the formation of WSs is partially explained by the degree of hypothermia that animals experience during long periods of fatal or nearly fatal energy-calorie deficit, induced by both endogenous (drop in thermogenic capacity) and exogenous (environmental temperatures) factors. Furthermore, experimental studies in animal models also suggest that stress induction could be associated with a higher incidence of superficial gastric bleeding and erosions. Specifically, rats exposed to low environmental temperatures showed fewer WS-like mucosal changes if allowed to bite vigorously while forcefully restrained.^28^ In contrast, rats that were hypothermic and submerged in cold water demonstrated a larger number of superficial gastric ulcerations if kept conscious during the experimental procedure.^17^ After a short period of fasting, rats developed WS-like changes of the gastric mucosa simply after the forceful restraint in a metal mesh as an experimental stressor.^11^ In contrast, unconscious rats that were exposed to environmental cold temperature to induce fatal hypothermia did not show WS-like mucosal changes at postmortem examination.^5^ In summary, the stress appeared to be a dominant cofactor in determining WS-like changes in the gastric mucosa, sufficient to induce mucosal bleeding in experimental models, even without hypothermia. Thus, we postulate that the high prevalence of WSs in the gastric mucosa of starved dogs and cats is the result of the combined effect of a drop in core body temperature during severe calorie deficit and, most importantly, the presence of concomitant and prolonged stress due to the total food deprivation and forceful confined isolation.

Despite the overall number of WSs in the mucosa being rarely as prominent as in reported cases of hypothermia in humans,^7,18,21,23,26^ the leakage of hemoglobin from gastric hemorrhages is probably established and occurs over long periods before death, at least superior to the minimum transit intestinal time; since golden granular material, identical to the one composing the WS of the stomach, is visible along the luminal surface of the intestine under light microscopy and black discolored digesta are noted throughout the distal gastrointestinal system and soiling the perianal fur. Anecdotic observations of crime scene photographs where the cadavers of starved dogs have been retrieved, in our personal experience, often showed the presence of vast areas of soiling with black feces, thus supporting the hypothesis that spillage of hemoglobin from gastric WSs surpassed the minimum intestinal transit time and lasted for a much longer time than just the agonic stage.

Due to the high specificity of WSs in canine and feline starvation, we strongly advocate the implementation of delicate but thorough washing of the gastric mucosa to document the possible presence of WSs in every postmortem examination of dogs and cats that appear in a poor state of nutrition. In scientific human literature, there is no mention of dark black discoloration of the gastrointestinal content. In contrast, the occurrence of WS is often interpreted as an agonal change restricted to the peri-mortal period.^27^ Our cases, instead, frequently presented dark brown to black discoloration of the intestinal content and feces. The latter element could assist in suspecting a death by starvation in emaciated dogs with black discolored feces, even if a complete postmortem remains the gold-standard approach to rule out other sources of proximal gastrointestinal bleeding.

Since the consistent black discoloration of the gastrointestinal content distally to the stomach strongly suggests the existence of gastric WSs and anticipates a diagnosis of fatal starvation, a detailed inspection of the gastric mucosa and documentation of WSs should be included within the routine forensic postmortem protocol. The identification of these spots in canine and feline starvation could serve as a model for comparative forensic pathology, mirroring observations traditionally associated with hypothermia deaths in humans.
